# Genetic modification of intractable bacterial clones by heat shock-facilitated phage transduction

**DOI:** 10.1016/j.crmeth.2026.101406

**Published:** 2026-04-20

**Authors:** Lukas Schulze, Jens Stahl, Nastassia J. Knödlseder, Sophia Krauss, Theresa Harbig, Kay Nieselt, Holger Brüggemann, Bernhard Krismer, Andreas Peschel

**Affiliations:** 1 Department of Infection Biology, Interfaculty Institute of Microbiology and Infection Medicine, University of Tübingen, Tübingen, Germany; 2 Cluster of Excellence EXC 2124, Controlling Microbes to Fight Infections, Tübingen, Germany; 3 German Center for Infection Research (DZIF), Partner Site Tübingen, Tübingen, Germany; 4 Department of Biomedicine, Aarhus University, Aarhus, Denmark; 5 Department of Medicine and Life Sciences, Universitat Pompeu Fabra, Barcelona, Spain; 6 Institute for Bioinformatics and Medical Informatics, University of Tübingen, Tübingen, Germany

**Keywords:** bacteriophages, staphylococci, transduction, restriction-modification, restriction barrier, genetic manipulation, heat shock

## Abstract

Increasing recognition of commensal bacteria as essential for microbiome integrity and pathogen exclusion underscores the urgency of molecularly characterizing commensal interactions. However, many commensals cannot be transformed using available methodologies due to barriers imposed by restriction-modification (RM) systems. We developed a method for introducing plasmid DNA into otherwise intractable non-*Staphylococcus aureus* (NAS) staphylococci, important commensals of the human nasal and skin microbiomes, via phage transduction. We demonstrate that exposing recipient bacteria to a pulse of elevated temperature prior to phage exposure renders NAS isolates effectively and transiently amenable to transduction. Transduction of NAS mutants lacking RM systems did not respond to heat shock, indicating that transient deactivation of RM enzymes enables transduction. Our method also facilitates the transduction of representatives from other Bacillota and Actinomycetota taxa, suggesting that this approach will support research on diverse bacterial groups across a range of ecosystems.

## Highlights

• Restriction-modification (RM) systems hinder the studying of many bacteria
• RM systems can be inactivated by a short heat shock
• Heat-shocked bacteria become genetically accessible
• Transduction plus heat shock enables manipulation of otherwise inaccessible bacteria

## Motivation

Many strains of staphylococci are resistant to genetic manipulation. This widespread challenge prevents scientists from studying the metabolism, pathogenicity, and capabilities of most commensal and environmental bacteria. To address this, we developed a method that successfully introduces DNA into previously inaccessible strains by combining a brief heat shock with phage transduction, which temporarily inactivates DNA-defense systems that prevent transduction.

## Graphical abstract

**Figure:**
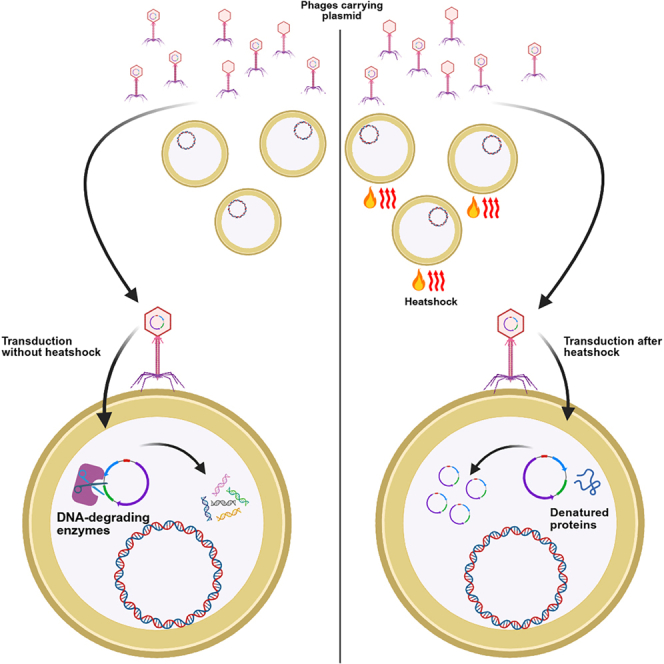

## Teaser

The majority of bacteria remain genetically inaccessible, significantly hindering the study of their metabolism, pathogenicity, and general capabilities. Schulze et al. describe a method that combines temporary inactivation of DNA-defense systems by heat shock with phage transduction to introduce DNA into otherwise inaccessible bacteria.

## Introduction

Staphylococci are prominent members of the human microbiome, including both, commensals and facultative pathogens, that impact on health or disease of their host in multiple ways.^1^^,^^2^^,^^3^ Much attention has been devoted to the coagulase-positive species *Staphylococcus aureus*, which is an opportunistic pathogen, responsible for severe soft tissue infections, bacteraemia, endocarditis, and many other types of infection.^4^^,^^5^ In contrast, infections caused by non-*S*. *aureus* (NAS) staphylococci are usually less severe, but they have gained increased attention in recent years, because NAS such as *Staphylococcus epidermidis* are major causes of catheter or prosthetic joint-associated infections.^6^^,^^7^ On the other hand, many isolates from NAS species such as *S. epidermidis*, *Staphylococcus lugdunensis*, or *Staphylococcus capitis* have also been reported to protect their host from *S. aureus* colonization by the production of *S. aureus*-eliminating antimicrobial secondary metabolites such as bacteriocins.^8^^,^^9^^,^^10^^,^^11^ Some bacteriocins have even been found to amplify the immune response or to act synergistically with host-derived antimicrobial peptides, demonstrating the fine-tuned interplay between the human host and its staphylococcal commensals.^12^^,^^13^

Genetic tractability is a prerequisite for studying the lifestyle and the phenotypic traits of bacteria, including those of staphylococci. To this end, a plethora of methods for bacterial genetic transformation and manipulation has been established throughout the last decades. While chemical transformation and conjugation are favorable methods for transferring genetic material into many bacterial species,^14^^,^^15^^,^^16^^,^^17^ such methods are not applicable to staphylococci, as natural competence is usually not observed and conjugation is rare in this genus.^18^^,^^19^ However, methods for phage-mediated DNA transfer, known as transduction,^18^^,^^19^ as well as the development of protocols for transformation by electroporation^20^ have greatly advanced the ability to study and work with many staphylococcal strains.

Nevertheless, studying staphylococci remains challenging, as especially NAS possess strong and diverse barriers for the introduction of foreign DNA, which impede genetic manipulation and render many strains entirely inaccessible to molecular research. Bacteria can encode four different types of restriction-modification (RM) systems, types I, II, III, or IV. These systems consist of various combinations of restriction endonucleases (REase), DNA-methyltransferases (MTase), and sequence specificity-conferring proteins. Type I systems consist of all three proteins while the type II and type III systems consist only of REase and MTase, and type IV systems of just a single REase.^21^^,^^22^^,^^23^^,^^24^ Previously, all four different RM types have been described in staphylococci.^25^^,^^26^^,^^27^^,^^28^^,^^29^ Importantly, individual strains of the same species often express multiple different RM-systems, with different sequence specificities.^30^ In addition to RM systems, staphylococci can express clustered regularly interspaced short palindromic repeat (CRISPR)-Cas systems, a sophisticated form of bacterial adaptive immunity, for the detection and degradation of foreign DNA.^31^^,^^32^

To circumvent these DNA immunity systems and prevent the degradation of foreign DNA by staphylococcal recipient strains, various strategies have already been published. For example, a short heat shock prior to electroporation significantly increased the transformation efficiency in *S. aureus* and *Staphylococcus carnosus*, probably by denaturing RM proteins transiently.^33^^,^^34^ As this methodology has only been validated for those two species with varying, strain-dependent success, a more general procedure, based on plasmid artificial modification (PAM) has been developed. Here, transformation efficiency of recipient strains with a strong RM barrier is enhanced by passaging the plasmid through a specifically engineered *E. coli* host. This intermediary host expresses the RM system-specific methylase to modify the transferred DNA with a suitable methylation pattern that protects it from degradation by the recipient strain.^35^^,^^36^ However, this approach is time and labor-intensive, as the modification system of the strain of interest must be identified, cloned, and expressed in *E. coli*. Subsequently, the methylated plasmid must be isolated from this modified strain for electroporation into the staphylococcal strain of interest. Moreover, this approach is usually not efficient for recipients expressing more than one RM system and therefore not suitable for most NAS isolates, which usually have more than one RM system.

We describe a new technique, which combines heat shock and transduction by a suitable phage, to enable the genetic manipulation of previously intractable staphylococcal strains and species. Our experimental data suggest that temporary inactivation of the restriction systems by the heat shock enables the successful introduction of foreign DNA into these strains. The new method is applicable for different plasmids, *Staphylococcus* species, and transducing phages. Additionally, it is also of potential value for the skin commensal *Cutibacterium acnes*, and it was effective in non-staphylococcal Bacillota, such as *Bacillus* sp. or *Listeria* sp., thereby demonstrating its potential benefit for the wider microbiological community.

## Results

### Identification of systems protecting from invading DNA

**Figure 1:**
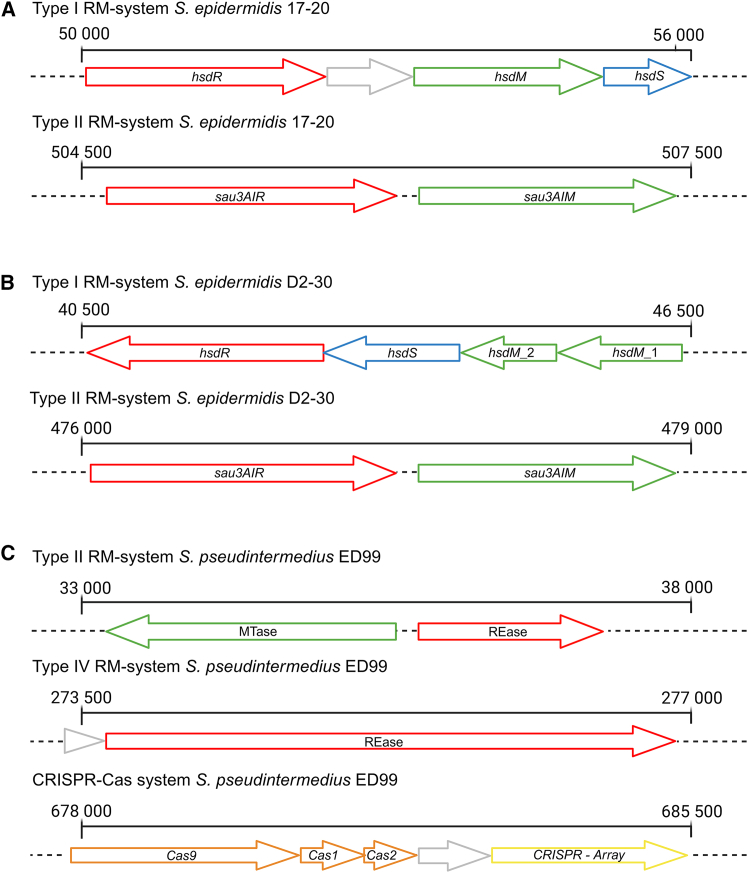
Overview of the RM and CRISPR-Cas systems identified via PADLOC (v2.0.0) for strains *S. epidermidis* 17-20, *S. epidermidis* D2-30, and *S. pseudintermedius* ED99 Methyltransferase genes are highlighted in green; genes mediating sequence specificity of the RM system are highlighted in blue, and genes encoding endonucleases are shown in red. Genes with unknown function are shown in gray. Numbers indicate the position of the systems within the respective genomes. (A) Two RM systems were identified for *S. epidermidis* 17-20. One type I system, comprised of the genes *hsdR*, *hsdS*, and *hsdM*. In addition, the cluster contains a gene with unknown function. The other system is the type II sau3AI system with two genes, for the restriction endonuclease (*sau3AIR*) and the methyltransferase (*sau3AIM*). (B) Two RM systems were identified in *S. epidermidis* D2-30. The first is a type I RM system, containing the genes *hsdR*, *hsdS*, and *hsdM*. The system contains two *hsdM* copies (*hsdM*_1 and *hsdM*_2). The second system is a type II RM system, identical to the sau3AI system shown for *S. epidermidis* 17-20. (C) *S. pseudintermedius* ED99 possesses a type II, as well as a type IV RM system in addition to its CRISPR-Cas system. The type II system consists of a methyltransferase and a restriction endonuclease gene. The type IV system is composed of a single restriction endonuclease gene. The CRISPR-Cas cluster consists of three CRISPR-associated (Cas) endonuclease genes; *Cas9*, *Cas1*, and *Cas2*. The cluster also contains a CRISPR array with multiple spacers and a repeat sequence.

Genetic manipulation of colonizing or infecting *Staphylococcus* isolates remains a major obstacle, mostly because of barriers that prohibit the introduction of recombinant DNA via transformation or transduction. We selected the two *S. epidermidis* strains, 17-20 and D2-30, isolated from human nasal microbiomes, and the *S. pseudintermedius* strain ED99 (all strains are summarized in Table S1) from canine skin infection,^37^ which could not be transformed or transduced with standard methods, to elucidate more effective ways for the introduction of plasmid DNA. The genomes of the three strains were analyzed for the presence of potential RM and CRISPR-Cas systems using the “prokaryotic antiviral defence locator” (PADLOC).^38^ *S. epidermidis* 17-20 encoded two RM systems (Figure 1A), a type I and a type II RM system, composed of three and two genes, respectively. The genes of both systems were analyzed with the REBASE database,^39^ to identify potential DNA recognition motives. The type I and type II systems were predicted to use the recognition sequences “GAGN_7_TAC” or “GWAGN_6_TTTA” and “GATC,” respectively.

*S. epidermidis* D2-30 also encoded one type I and one type II system (Figure 1B) with the putative recognition sequences “GAAYN_5_TGC” and “GATC,” respectively. The type II RM system proteins of the two *S. epidermidis* strains showed 100% identity to each other and to the Sau3AI system proteins of other *S. epidermidis* strains. Similarly, the identity was 70% for the restriction enzyme Sau3AIR and 77% for the methyltransferase Sau3AIM to those of *S. aureus*,^40^ which also uses the GATC recognition sequence.

*S. pseudintermedius* ED99 was found to encode a type II RM system with predicted recognition sequence “CTRYAG,” a type IV restriction endonuclease with unclear specificity, and a CRISPR-Cas array (Figure 1C). The CRISPR-locus carried a Cas9 endonuclease, a CRISPR-associated nuclease Cas1, a CRISPR-associated nuclease Cas2, and a CRISPR array containing multiple spacers and the repeat sequence “GTTTTAGCACTATGTTTATTTAGAAAGAGGTAAAAC,” indicating the presence of a presumably fully functional CRISPR-Cas system. An overview of all relevant defense systems, which may explain the difficulties in transformation of the three strains, was acquired from the PADLOC webserver and is summarized in Table S2.

### A brief heat shock renders test strains susceptible to phage transduction

Previously established protocols for electroporation of staphylococci^41^^,^^42^ were not successful for the transformation of the three test strains with different plasmids (see Table S3). A heat shock, applied to the competent cells prior to electroporation, as previously suggested by Löefblom et al.,^33^ did also not result in any transformants. Since phage transduction is known to be an effective alternative for introducing DNA into staphylococci, we investigated whether the plasmids could be transferred into these strains via phage transduction. Phage ΦE72 was chosen for initial experiments with *S. epidermidis* D2-30 and 17-20, as this phage has been shown to infect bacteria of the species *S. epidermidis.*^43^ Because ΦE72 has also been found to bind to *S. pseudintermedius* ED99,^44^ it was analyzed for its capacity to transduce ED99. However, no successful transduction occurred in our experiments with any of the three strains, using the standard method.

**Figure 2:**
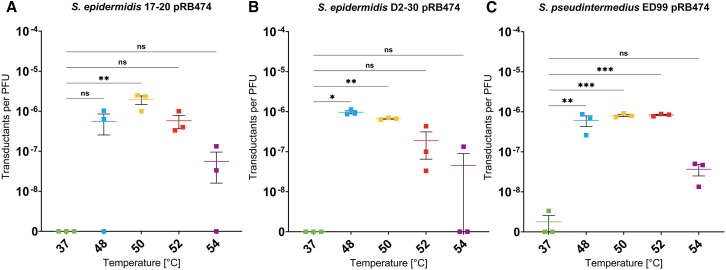
Heat shock transduction of different strains using plasmid pRB474 As control, cells were not heat shocked but incubated for 2 min at the regular growth temperature of 37°C. Transductions were carried out using phage ΦE72, propagated in *S. epidermidis* 1457, carrying plasmid pRB474. (A) Heat shock transduction of *S. epidermidis* 17-20 wild type with phage ΦE72 carrying plasmid pRB474. (B) Heat shock transduction of *S. epidermidis* D2-30 with phage ΦE72 carrying plasmid pRB474. (C) Heat shock transduction of *S. pseudintermedius* ED99 with phage ΦE72 carrying plasmid pRB474. Transductants per PFU shown on *y* axis in logarithmic scale at the different transduction temperatures. Data represent the mean of three independent biological replicates (*n* = 3) ± SEM. Statistical analysis was performed via one-way ANOVA with Dunnett’s multiple comparison test (using the 37°C condition as reference). ns, not significant; ∗*p* < 0.05; ∗∗*p* < 0.01; ∗∗∗*p* < 0.001. See also Figures S1 and S2.

**Figure 3:**
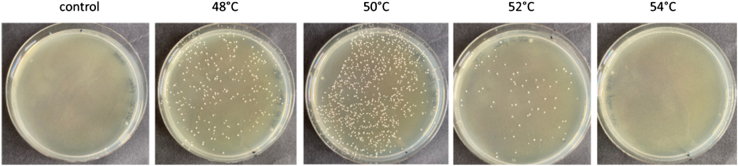
TSA plates, (supplemented with chloramphenicol) 48 h after transduction of *S. epidermidis* D2-30 with the plasmid pBASE6. *S. epidermidis* D2-30 was transduced with phage ΦE72, propagated on *S. epidermidis* 1457 carrying plasmid pBASE6 The picture shows a single representative transduction replicate under standard condition (control) or increased temperatures.

We reasoned that a heat shock step that has been helpful for improving the yields of electroporation in certain *Staphylococcus* strains might also increase the efficacy of phage transduction. To test this possibility, cells of the three test strains were heat-shocked for 2 min at 48°C, 50°C, 52°C, or 54°C prior to the addition of phage lysates. These temperatures were chosen according to the previous report of Löefblom et al.^33^ We found indeed that the new method generated successfully transduced bacterial cells for all three strains. The significant increase in the transduction efficiency, measured in transductants per plaque-forming units (PFU), was already observed when the cells were heat-shocked at temperatures ≥48°C (Figures 2 and 3). However, the heat shock at 54°C led to markedly lower transduction efficiency compared to temperatures between 48°C and 52°C (Figure 2). The optimal heat shock temperature was dependent on the recipient strain and the plasmid used. For *S. epidermidis* 17-20, a temperature of 50°C yielded the highest transduction efficiency for plasmid pRB474 (Figure 2A), while 52°C was optimal for pBTn (Figure S1). In the case of *S. epidermidis* D2-30, the 48°C heat shock led to the highest efficiency with all plasmids (Figures 2B and S2), except pBASE6, for which 50°C was slightly more efficient (Figure S2). Interestingly, little to no difference in transduction efficiency was observed for *S. pseudintermedius* ED99 with all plasmids used between 48°C and 52°C, but 54°C also decreased the transduction efficiency (Figure 2C).

### Transduction shows enhanced efficiency in comparison to transformation by electroporation

We investigated why heat shock-supported transduction but not heat shock-supported electroporation was effective at introducing DNA in our test strains. We hypothesized that either the combined stress of heat shock and electroporation is too challenging for the cells, or that transduction represents generally a more effective method, resulting in more clones per applied DNA amount. Investigating the first hypothesis, we challenged electrocompetent cells of *S. aureus* RN4220 by standard electroporation, a heat shock at 52°C, or a combination of both. While we found that the two individual conditions led to a significant decrease in viable cell numbers, the combination of both reduced the viable cells count even further, by more than 100-fold (Figure S3) leaving less than 1% of the applied cells viable and capable of DNA uptake.

For the evaluation of efficiencies, we performed transformation and transduction experiments in parallel. Plasmid pRB473 was isolated from *S. aureus* RN4220 for electroporation, and this strain was also used to generate a lysate of phage Φ11. When using the isolated plasmid for electroporation and the generated phage lysate for transduction of *S. aureus* RN4220 wild type (without heat shock), we found a significant difference in efficiency, shown as number of transformants/transductants per molecule of provided plasmid DNA (Figure S3). The proportion of plasmid bearing phages (transducing particles) and the resulting number of transductants per applied molecule of DNA was significantly higher in comparison to the number of transformants per DNA molecule during transformation.

### The duration of the heat shock influences transduction efficiency

**Figure 4:**
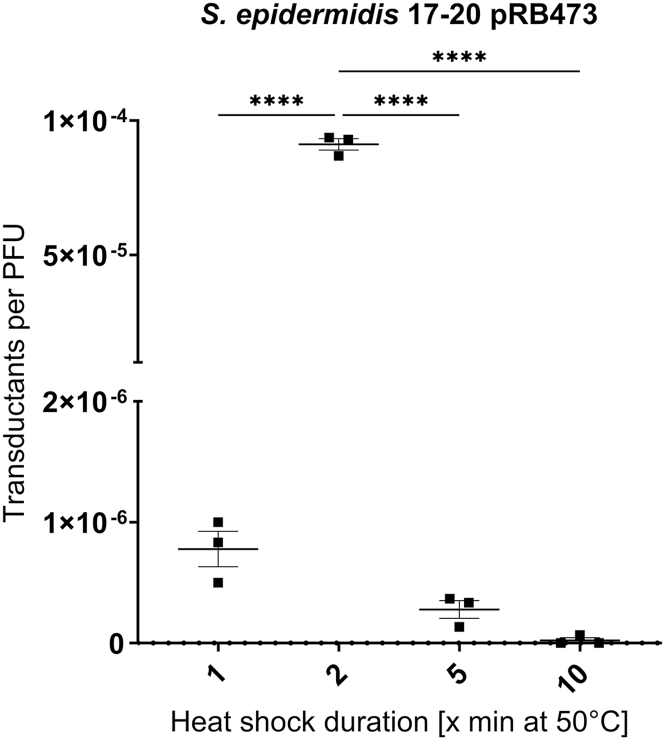
Influence of heat shock duration on transduction efficiency Wild-type cells of *S. epidermidis* 17-20 were heat shocked at 50°C for 1, 2, 5, or 10 min prior to transduction with phage ΦE72 carrying plasmid pRB473. Phage ΦE72 was propagated in *S. epidermidis* 1457 WT carrying plasmid pRB473 to generate the lysate used for transduction. Transductants per PFU shown on *y* axis in logarithmic scale at different heat shock durations. All data represent *n* = 3 individual replicates and are shown as mean ± SEM. Statistical analysis was performed using one-way ANOVA with Dunnett’s multiple comparison test. The 2-min time point was used as reference. ∗∗∗∗*p* < 0.0001.

To further optimize the transduction conditions for staphylococci, the heat shock duration was varied between 1 and 10 min, using *S. epidermidis* 17-20 at the optimal temperature of 50°C as shown above (Figure 2A). We found that our initially chosen duration of 2 min yielded the highest increase in transduction efficiency in comparison to the shorter or longer time periods (Figure 4). A heat shock of 1 or 5 min still resulted in successful transduction of the strain, although at much lower efficiency, while only residual transductants could be found after the 10-min heat shock, indicating that this duration is too harsh for the cells to be efficiently transduced.

### Inactivation of restriction enzymes renders the heat shock prior to phage transduction dispensable

**Figure 5:**
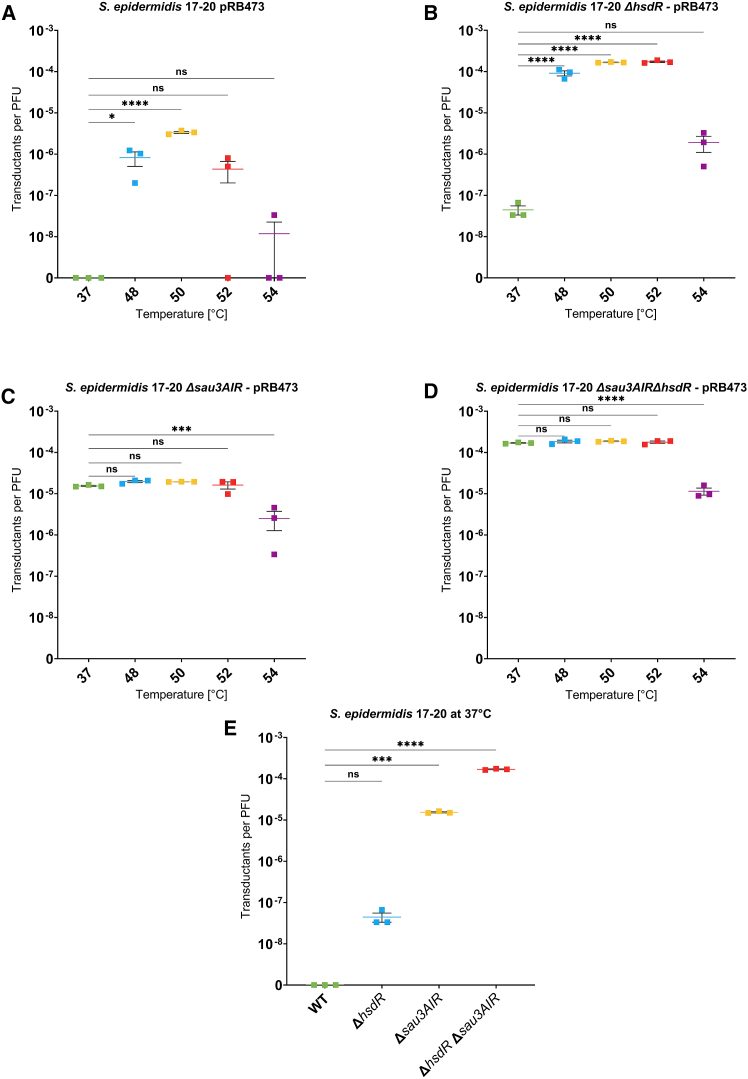
Heat shock transduction of *S. epidermidis* 17-20 and three restriction-deficient deletion mutants All experiments were performed using plasmid pRB473 and phage ΦE72. As control, cells were not heat shocked but incubated for 2 min at the regular growth temperature of 37°C. (A) Transduction of *S. epidermidis* 17-20 wild type with phage ΦE72 carrying plasmid pRB473. (B) Heat shock transduction of *S. epidermidis* 17-20Δ*hsdR* with phage ΦE72 and plasmid pRB473. (C) Heat shock transduction of *S. epidermidis* 17-20 Δ*sau3AIR* with plasmid pBR473 via phage ΦE72. (D) Heat shock transduction of *S. epidermidis* 17-20 Δ*hsdR*Δ*sau3AIR* via phage ΦE72 with plasmid pRB473. Transductants per PFU shown on *y* axis in logarithmic scale at the different transduction temperatures. (E) Transduction efficiency of *S. epidermidis* 17-20 wild type and its restriction deficient mutants at 37°C. Transductions of plasmid pRB473 via phage ΦE72, which was propagated in *S. epidermidis* 1457 carrying pRB473. Transductants per PFU are shown on the *y* axis on logarithmic scale after transduction at 37°C. All data represent *n* = 3 individual replicates and are shown as mean ± SEM. Statistical analysis was performed via one-way ANOVA with Dunnett’s multiple comparison test (37°C as reference). ns, not significant; ∗*p* < 0.05; ∗∗*p* < 0.01; ∗∗∗*p* < 0.001; ∗∗∗∗*p* < 0.0001.

We hypothesized that the restriction systems encoded by the recipient strains are the reason for the inability to transduce the strains with conventional methods, and that the heat shock may have led to temporary inactivation of restriction endonucleases. To test this assumption, we created *S. epidermidis* 17-20 mutants lacking the type I RM system (Δ*hsdR*), the type II RM system (Δ*sau3AIR*), or both (Δ*hsdR*Δ*sau3AIR*) by homologous recombination (primers used see Table S4). The transduction with prior heat shock of the mutant strain panel showed higher transductant numbers, also at the control temperature of 37°C, in comparison to the wild type (Figure 5). As observed before, a heat shock of 48°C–52°C led to a significant increase in transduction efficiency in *S. epidermidis* 17-20 wild type, whereas 54°C was less effective (Figure 5A). While there was still a significant increase in transduction efficiency in *S. epidermidis* 17-20 Δ*hsdR* by the heat shock (Figure 5B), we found that the heat shock only slightly increased transduction efficacy in *S. epidermidis* 17-20 Δ*sau3AIR* (Figure 5C) or in the double mutant *S. epidermidis* 17-20 Δ*hsdR*Δ*sau3AIR* (Figure 5D). The transduction efficiency decreased after the heat shock at higher temperatures, probably because it led to damage to the cells. Comparison of transduction efficiencies at 37°C of the *S. epidermidis* 17-20 wild type and its RM-system deficient mutants confirmed that loss of RM-competence led to a significant increase in transducability at the control temperature of 37°C (Figure 5E).

### Increased transduction efficiency by heat shock is only transient

**Figure 6:**
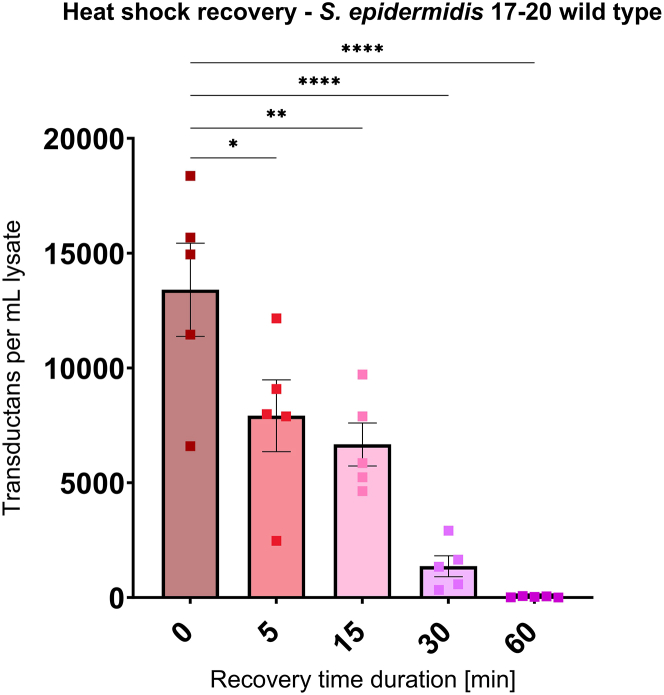
Heat shock recovery assay after heat treatment for 2 min at 50°C prior to transduction *S. epidermidis* 17-20 wild type was transduced with phage ΦE72, which was propagated in *S. epidermidis* 1457 carrying plasmid pRB473. After the heat treatment cells were allowed to recover in TSB for 5, 15, 30, or 60 min before addition of phage ΦE72 lysate and subsequent transduction (for *t* = 0 min cells were transduced directly after heat treatment without recovery time to provide a reference value for successful transduction). Transductants per mL lysate are shown on *y* axis, plotted after the different recovery times. All data represent *n* = 5 individual replicates, and the mean ± SEM is shown. Statistical analysis was performed via one-way ANOVA with Dunnett’s multiple comparison test (recovery time of *t* = 0 min as reference). ∗∗*p* < 0.01; ∗∗∗*p* < 0.001; ∗∗∗∗*p* < 0.0001.

To determine how long the transduction capacity of the test strains persists after the heat shock, the *S. epidermidis* 17-20 wild type was heat-shocked at 50°C, and cells were either directly exposed to the phage lysate (*t* = 0 min) or after 5, 15, 30, or 60 min of regeneration in broth at 37°C before transduction. While many transductants were observed at *t* = 0 min, a gradual decrease in efficiency was observed with increasing recovery time. After 60 min of recovery, no transductants could be found any more, indicating that the increased transduction competence was only transient (Figure 6).

### Heat shock increases transduction efficiency also in other bacterial species and genera

We tested the new transduction method on clinical *S. aureus* strains but found no isolates that were difficult to transduce with phage Φ11 propagated on another *S. aureus* strain such as *S. aureus* RN4220. As the transduction rate was already high, the heat shock protocol did not further improve efficiencies.

**Figure 7:**
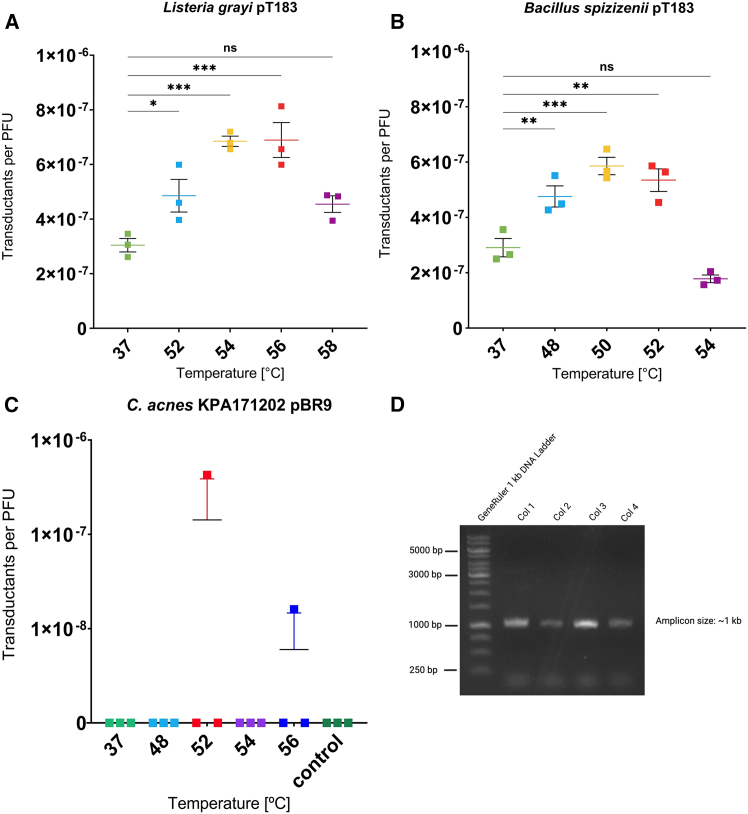
Influence of heat shock on transduction efficiency in *Bacillus spizizenii* and *Listeria grayi* using phage Φ11 carrying plasmid pT183 and heat shock transduction of *C. acnes* KPA171202 using phage PAD20 carrying plasmid pBR9 Phage Φ11 was propagated in *S. aureus* RN4220 carrying plasmid pT183 to generate the phage lysate, while phage PAD20 was propagated in *C. acnes* SLST A1 carrying pBR9. (A) Influence of the heat shock on transduction efficiency for *L. grayi* with phage Φ11 carrying plasmid pT183. Cells were heat shocked for 2 min at the indicated elevated temperatures or incubated at 37°C as control. (B) Heat shock transduction of *B. spizizenii* with plasmid pT183 via phage Φ11. Bacteria were heat shocked at the indicated temperatures for 2 min or incubated at 37°C as control. All data are shown as means of three independent biological replicates (*n* = 3) ± SEM. Statistical analysis was performed via one-way ANOVA with Dunnett’s multiple comparison test (37°C set as reference for comparison). ns, not significant; ∗*p* < 0.05; ∗∗*p* < 0.01; ∗∗∗*p* < 0.001. (C) *C. acnes* KPA171202 was heat shocked for 2 min at 37°C, 48°C, 50°C, 52°C, 54°C, or 56°C. Transductants per PFU are shown on logarithmic scale on the *y* axis. Furthermore, denatured phage PAD20 (control) was added as negative-control and treated the same (transduction tested at all temperatures with identical results, only three datapoints are shown). (D) Colony PCR of some *C. acnes* KPA171202 clones generated via heat shock transduction. Lane 1: GeneRuler 1 kb DNA ladder; lanes 2–5: representative colonies tested (expected fragment size for successful uptake of plasmid pBR9 ∼1 kb)

To evaluate if the developed protocol for highly efficient transduction is applicable also to difficult-to-transform bacteria other than staphylococci, we studied transduction of plasmid DNA from *S. aureus* RN4220 to *Bacillus spizizenii* and *Listeria grayi*. Additionally, we tested the method for transduction of *Cutibacterium acnes*, which is known to possess a strong RM-barrier that hampers efficient genetic manipulation.^45^^,^^46^^,^^47^ Both, *L. grayi* and *B. spizizenii*, could be transduced at reasonable efficiency with phage Φ11 carrying the staphylococcal plasmid pT183 even at 37°C without a heat shock (Figure 7). Nevertheless, the application of a heat shock increased the transduction efficiency by up to ∼120% in *L. grayi* (Figure 7A) or ∼100% in *B. spizizenii* (Figure 7B), respectively. The two species differed in the optimal heat shock temperature, which was found to be 54°C to 56°C or 50°C for *L. grayi* or *B. spizizenii*, respectively. For *Cutibacterium acnes* strain KPA171202, we found no transductants with the previously described phage PAD20,^45^ using standard procedures without a heat shock. This is in line with prior studies, where this negative result was attributed to the active RM barrier (type IIIB) of strain KPA171202, as *C. acnes* strains lacking this type IIIB RM system can be generally accessed via PAD20.^45^ However, the application of a 2-min heat shock in the range of 52°C–56°C allowed successful transduction in one out of three samples, each one for the 52°C and the 56°C condition. This finding suggests that the method can be applied also to RM-competent *C. acnes*, although further improvements of the transduction protocols are necessary, to obtain consistent results (Figure 7C). The presence of the correct plasmid in the transductants was additionally confirmed in some of the resulting clones via PCR (Figure 7D).

## Discussion

Staphylococci represent key players in the human skin and upper respiratory microbiota, and they have multiple ways to affect health and disease of their host.^4^^,^^48^^,^^49^^,^^50^ However, while the frequent pathogen *S. aureus* has been studied extensively, the molecular characterization of commensal staphylococci remains challenging. These challenges mainly result from difficulties in genetic manipulation, probably because of the prevalence of one or more foreign DNA-degrading mechanisms in these bacteria. Classical RM systems have been studied for decades, and the presence of all four types of RM systems has been confirmed in staphylococci.^29^^,^^40^^,^^51^ More recent studies have found that some staphylococcal clones also encode CRISPR-Cas systems, which may contribute to the barrier, even though they occur in only a minority of staphylococcal isolates.^52^^,^^53^^,^^54^

Since the first discovery of bacterial transduction in *E. coli* by Lederberg et al. in the 1950s,^18^ the adaptation of the method for the genus *Staphylococcus*^19^ and the establishment of DNA transfer to staphylococci via electroporation,^20^ significant advances have been made, enabling the genetic manipulation of various staphylococcal species. These include the optimization of electroporation protocols,^33^^,^^42^ identification and use of novel transducing phages,^43^^,^^55^ generation of PAM systems,^56^^,^^57^ the use of specifically designed CRISPR-Cas systems,^58^ or stealth-by-engineering approaches, hiding DNA from recognition.^59^ However, many of these methods are only suitable for some bacterial strains, or they remain very labor-intensive. Despite all these efforts, many *Staphylococcus* isolates remain difficult or even impossible to manipulate. The work presented here aims at providing a method that overcomes transformation barriers even in the most refractory strains. Due to its easy, fast, and cost-effective use, we are confident that our method will advance the study of so far inaccessible bacteria, provided that transducing phages are available.

Our experimental data show that a heat shock applied to bacteria prior to exposure to different transducing phages, including the *S. epidermidis*-infecting ΦE72 and the *S. aureus*-infecting Φ11, led to a significant increase in transduction efficiency by, in some cases, multiple orders of magnitude. We wondered why the combination of heat shock plus transduction but not heat shock plus transformation allowed us to introduce DNA into these otherwise inaccessible strains. Comparing the viability of electrocompetent *S. aureus* RN4200 after heat shock, electroporation or both, indicated that while both stresses impact viability already, combining both methods reduced the number of viable cells even more strongly, by >99%, highlighting that the combination might be too detrimental to be effective for most bacteria. This finding is reminiscent of a previous study, indicating that heat treatment of *S. aureus* RN4220 strongly reduced the efficiency of electroporation and viability.^34^ Additionally, we compared the efficiency of transformation and transduction. Therefore, we analyzed the number of resulting colonies after electroporation or transduction relative to the amount of plasmid DNA used, in the form of “clones per plasmid copy.” The data show that the amount of DNA necessary for a singular DNA uptake event to occur in the recipient cells is substantially lower for transduction in comparison to transformation. In fact, the high affinity of phages for their bacterial receptors, as previously demonstrated for phage T5,^60^ can explain that most plasmid-bearing phage particles may deliver their cargo into bacteria, resulting in high numbers of transductants. In comparison, only a small percentage of applied free plasmid DNA might bind to and enter bacterial cells through the electric pulse during electroporation. As a result, electroporation usually has a lower efficiency compared to transduction. These results highlight that while electroporation represent a suitable method for inserting DNA into certain recipient cells, phage transduction offers a much more effective approach, in particular for difficult-to-transform bacterial isolates.

We found that application of this heat shock for varying periods significantly influences the efficiency, and 2 min seems to be the most efficient duration for the heat shock in staphylococci, which agrees with the previously published heat shock transformation protocol.^33^ However, it is possible that longer or shorter durations may be more efficient in other bacteria, which needs to be assessed in case-by-case attempts. We found that a temperature range between 48°C and 54°C is most suitable for heat shock transduction of staphylococci. However, higher or lower temperatures might be more beneficial for the transduction of other species, as found for the transduction of *L. grayi*. Notably, this method enabled us to transfer DNA into *C*. *acnes* strain KPA171202, where no transduction was possible without prior heat shock, or using a denatured phage lysate, bearing no intact phage particles. However, we observed a very low efficiency and difficulties with reproducibility in the transduction of *C*. *acnes* KPA171202 in comparison to all other tested bacteria. We assume that further optimization of heat shock duration, temperature, phage lysate titer, recovery conditions, etc. could help to improve the protocol for this species.

Elevated environmental temperatures are detrimental to cells due to their negative impact on protein stability.^61^^,^^62^ We assumed that the heat shock denatures restriction enzymes and therefore enables bacteria to take up and replicate foreign DNA, before degradation by newly synthesized or successfully refolded RM enzymes can occur. We found that both RM systems of *S. epidermidis* 17-20 impacted the ability of the strain to acquire foreign DNA, but that the type II system, Sau3AI, plays a dominant role, whereas the type I system has a subordinate function. However, a reduced transduction efficacy at the highest heat shock temperature of 54°C was obvious. We assume that incubation at this high temperature reduces the viability of the cells, since temperatures that impair the function of restriction enzymes would also damage other, essential cellular proteins. The cells need to express the plasmid-encoded antibiotic resistance determinant to survive in the presence of the antibiotic, but a shift to 54°C could damage various enzymes involved in transcription and synthesis of the resistance-conferring protein. This could in turn force the cells to focus on the protection and re-synthesis of essential cellular components and thereby leaving not enough time to reliably express the antibiotic resistance and as a result also reduce the overall efficiency of the method. This assumption is in accordance with previous findings, indicating that temperatures >50°C for a short time reduce the viability of staphylococcal cells.^63^^,^^64^

To cope with challenging environmental conditions, bacteria have evolved a large set of fine-tuned response-cascades, including the stringent, cold shock, and heat shock response (HSR), adjusting cellular processes to altered demands.^65^^,^^66^ The HSR induces various mechanisms at elevated temperatures involved, for instance, in repair of damaged DNA, stabilization of mRNA, degradation of misfolded proteins, as well as protection of proteins from damage or degradation by chaperones.^66^^,^^67^^,^^68^^,^^69^ We observed a gradual decrease in transduction efficiency upon heat shock over time, suggesting that the restriction mechanisms are restored within 1 h by the HSR or by *de novo* synthesis of restriction endonucleases.

Our heat shock transduction protocol was also effective in other bacterial genera. While *B. spizizenii* and *L*. *grayi* were also transducible using our standard protocol, a significant increase in transduction efficiency was found after application of a heat shock for both species, albeit at other optimal temperatures as for staphylococci. Thus, our method can be useful for a variety of bacterial species, but the height and duration of the heat shock will need to be optimized for each species.

In summary, our work demonstrates that a short heat shock applied to staphylococci and various other bacteria, prior to transduction, significantly increases transduction efficiency and thereby enables the work with otherwise genetically inaccessible strains.

### Limitations of the study

We also found a strong increase in transduction efficiency of *S. pseudintermedius* ED99, which, in addition to a type II and a type IV RM system, also encodes a CRISPR-Cas system. It remains to be analyzed if inactivation of this CRISPR-Cas system may contribute to the heat shock mediated increase of *S. pseudintermedius* transduction. While transduction of *C. acnes* using the herein described method was partially successful, problems in continuous reproducibility highlight that adaption of the protocol for the respective genus/species used for transduction must be evaluated experimentally.

## Resource availability

### Lead contact

Any requests for additional information, mentioned resources, or materials should be sent to and will be provided by the lead contact, Bernhard Krismer (b.krismer@uni-tuebingen.de).

### Materials availability

Any material and information used for this study are available from the lead contact upon request.

### Data and code availability

• Bacterial genomes were deposited at NCBI and are available under accession numbers GenBank: CP186575 and GenBank: CP185372 for *S. epidermidis* 17-20 and D2-30, respectively.
• No code has been generated in this study.
• Any further data are available from the lead contact upon request.

## Acknowledgments

The authors thank Vera Augsburger for excellent technical support and Janes Krusche and Christian Beck for experimental help. The authors also thank all members of the Peschel and Wolz lab for their help and advice. This work was supported by the German Research Foundation project SPP 2330 (ID 465126486) and PE 805/7-1 (ID 410190180) to A.P., the German Center for Infection Research to B.K. and A.P. (TTU HAI), and a LEO-Foundation grant (grant no. LF-VR-24-201017) to N.J.K. The authors acknowledge infrastructural support from the Cluster of Excellence EXC 2124 “Controlling Microbes to Fight Infections” (ID 390838134). The authors acknowledge support from the Open Access Publication Fund of the University of Tübingen.

## Author contributions

S.K. identified and characterized the strains; K.N. and T.H. sequenced the strains, assembled the genomes, and annotated them; B.K. assisted with experimental design, data interpretation, and writing the manuscript; J.S. and L.S. designed and performed the experiments, collected and analyzed the data, and wrote the manuscript draft; N.J.K. and H.B. designed experiments involving Cutibateria; N.J.K. performed and analyzed all Cutibacteria-related experiments; H.B. assisted with manuscript finalization; A.P. assisted with figure design, data interpretation, manuscript writing, and design and finalization; and A.P. and B.K. supervised the work.

## Declaration of interests

The authors declare no competing interests.

## STAR★Methods

### Key resources table

**Table:** 

REAGENT or RESOURCE	SOURCE	IDENTIFIER
**Bacterial strains and bacteriophages**		

Bacterial strains, Table S1	This paper	N/A
Bacteriophages, Table S1	This paper	N/A

**Chemicals, peptides, and recombinant proteins**		

Lysostaphin	Merck	Cat. No. L7386

**Critical commercial assays**		

Qiagen Plasmid MidiPrep Kit	Qiagen	Cat. No./ID. 12945
Ligation sequencing kit	Oxford Nanopore	SQK-LSK109
Native Barcoding Expansion 96	Oxford Nanopore	EXP-NBD196
Blunt/TA ligase master mix	New England Biolabs	Cat. No. M0367
Illumina Nextera DNA Flex library preparation kit	Illumina	Cat. No. 20018708
IDT for Illumina DNA/RNA UD indexes, Tagmentation	Illumina	Cat. No. 20091654
NEBNext Ultra II End Repair/dA – tailing module	New England Biolabs	Cat. No. E7546
AMPure XP	Beckman Coulter	Cat. No. A63881
Qubit dsDNA Quantification Kit, broad range	Thermo Fisher	Cat. No. Q32850
Qubit dsDNA Quantification Kit, high specificity	Thermo Fisher	Cat. No. Q32851
MiSeq Reagent Kit v2 (300-cycles)	Illumina	Cat. No. MS-102-2002

**Deposited data**		

Defense system identification, Table S2	PADLOC v 2.0.0	N/A
Genome *S. epidermidis* 17-20	NCBI	GenBank: CP186575
Genome *S. epidermidis* D2-30	NCBI	GenBank: CP185372

**Oligonucleotides**		

Plasmids, Table S3	This study	N/A
Knockout plasmid primers, Table S4	This study	N/A
Sequencing primers, Table S4	This study	N/A

**Software and algorithms**		

Graphpad PRISM version 10.0.0	Graphpad	https://graphpad.com/
MLST 2.0	Center for Genomic Epidemiology	https://cge.food.dtu.dk/services/MLST/
PADLOC (v2.0.0)	Jackson Lab, University of Otago	https://padloc.otago.ac.nz/padloc/
REBASE	New England Biolabs	https://rebase.neb.com/rebase/rebase.html
BLAST	NCBI	https://blast.ncbi.nlm.nih.gov/Blast.cgi
Biorender	Biorender	https://www.biorender.com
NEBio Calculator	New England Biolabs	https://nebiocalculator.neb.com/#!/dsdnaamt
Guppy (v4.1.1)	Oxford Nanopore https://community.nanoporetech.com/	https://community.nanoporetech.com/
bcl2fastq (v2.19.0.316)	GitHub	https://github.com/brwnj/bcl2fastq
Unicycler (v0.5.0)	Wick, R. et al.^70^	https://github.com/rrwick/Unicycler
Prokka (v1.14.6)	Seemann, T.^71^	https://github.com/tseemann/prokka
quast (v5.3.0)	Gurevich, A. et al.^72^	https://github.com/ablab/quast

### Experimental model and study participant details

#### Nasal bacteria isolation

Isolation of bacterial strains from the nose of healthy human volunteers was performed during a student’s practical course at the University of Tübingen. Phosphate buffer saline (PBS) soaked swabs were used by students to swab their own noses and samples were streaked out on Tryptic Soy Agar (TSA) plates. The plates were incubated for 48h at 37°C to allow for various bacteria to grow.

Students were informed about the intended use of the strains isolated from their samples and consent was given by the students. No further information regarding the specific association between strains and donor as well as any further donor information are available. The sample collection procedures were approved by the clinical ethics committee of the University of Tübingen (No. 109/2009 BO2) and oral consent was given by all students. Nasal swabs were taken exclusively from healthy adults. The sex of the participants was not collected.

#### Bacterial strains

All bacteria used in this work are listed in the Key Resource Table (KRT). All staphylococcal strains used in this work were grown in Tryptic Soy Broth (TSB; Oxoid, Germany) except for *S. epidermidis* 1457, which was grown in ‘Basic Medium’ (BM, 1% soy peptone, 0.5% yeast extract, 0.5% NaCl, 0.1% K_2_HPO_4_, and 0.1% Glucose). *Escherichia coli* was grown in lysogeny broth (LB-Lennox; Sigma-Aldrich). *Cutibacterium acnes* strains were grown in Brain Heart Infusion Broth (BHI; Condalab). For cultivation on plates, 1.5% agar (15 g L^−1^; BD) was added to the respective media, or 0.5% (5 g L-^1^) if soft-agar was prepared. If necessary, plates or liquid media were supplemented with the appropriate antibiotics at concentrations of 10 μg mL^−1^ (chloramphenicol; Sigma-Aldrich), 100 μg mL^−1^ (ampicillin; Carl-Roth), 12.5 μg mL^−1^ (tetracycline; Carl-Roth) or 10 μg mL^−1^ (erythromycin; Sigma Aldrich). For the preparation of day-cultures used in transduction assays or propagation, fresh medium was inoculated to OD_600_ = 0.1 using overnight cultures incubated for 16–20 h. All liquid cultures were grown shaking at 110 to 160 rpm and 37°C (New Brunswick Scientific – Innova44, Eppendorf) or incubated anaerobically when growing *Cutibacterium acnes* strains. Temperature was decreased to 30°C for incubation of the staphylococci if temperature-sensitive plasmids pBASE6 or pBTn were present. Plates were incubated stationary at 37°C, except for plates containing staphylococci harboring temperature-sensitive plasmid pBASE6 or pBTn, which were incubated at 30°C.

#### Bacteriophages

Phages used in this work are Φ187, ΦE72, Φ11 and PAD20. Propagation and lysate preparation for phage Φ187 were carried out using the bacterial strain *S. aureus* PS187ΔΔ (Δ*sauUSI*Δ*hsdR)* while phage ΦE72 was propagated in *S. epidermidis* 1457, phage Φ11 in *S. aureus* RN4220 and PAD20 was propagated on *C. acnes* skin isolate SLST type A1.

### Method details

#### Phage propagation and phage lysate preparation

To propagate the phages, the respective propagation strains were inoculated into liquid cultures and grown over night (16–20 h). The next day, cultures were diluted to OD_600_ = 0.1 in fresh medium and grown till OD_600_ = 0.4 was reached. CaCl_2_ was added to the culture at a final concentration of 4 mM to increase phage binding. Subsequently, lysate of the phage to be propagated was added (roughly 1/5 of the bacterial culture), and the mixture was incubated shaking at a reduced speed of 70 rpm and 37°C until the culture became clear (2–12 h). Lysed cultures were centrifuged for 10 min at 4,700 x g to pellet cell debris, and the resulting lysates were sterile filtered using a 0.22 μm filter (Millex; Merck).

Phage titers were determined by standard plaque formation assay. Therefore, the propagation strain was grown over night in liquid medium. The next day, TSA soft agar (for *S. aureus* PS187ΔΔ and RN4220), BM soft agar (for *S. epidermidis* 1457) or Brucella soft agar (for *C. acnes* SLST A1) were prepared. After cooling to 50°C, soft agar was inoculated with *S. aureus* PS187ΔΔ, *S. aureus* RN4220, *S. epidermidis* 1457 or *C. acnes* SLST A1 overnight culture to OD_600_ = 0.1, thoroughly mixed, and 5 mL poured onto pre-poured plates of either TSA, BM or BHI (depending on soft agar used). Lysates or propagations of the different phages were diluted in a dilution series to 10^−8^ with phage buffer. 10 μL of the phage suspension and the dilutions were spotted in triplicates on the soft agar containing the corresponding propagation strain and plates incubated ON at 37°C. The next day, individual plaques were counted and the phage titer calculated as PFU ∗ mL^−1^.

To prepare lysates for transduction assays, the same procedure was performed, using the propagation strain carrying the plasmid of interest. However, after addition of the phage to bacteria carrying a temperature-sensitive plasmid, the mixture was incubated shaking at 70 rpm and 30°C instead of the 37°C described above. Propagated phages and lysates were stored at 4°C until use. For transduction, only lysates with a phage titer of at least 5 × 10^8^ PFU mL^−1^ were used to obtain reproducible results.

For control reasons Phages propagated on *C. acnes* SLST A1 strain were denatured as follows. 1mL of previously sterile filtered (0.22 μm) Phage lysate was incubated with 6 μl Proteinase K (20 mg/mL) for 1 h at 37°, followed by heat denaturation for 20 min at 95°. Phage denaturation was confirmed by serial dilution on top agar plates. Denatured phages were used as an independent sample at all transduction temperatures and compared to non-denatured Phage samples in transduction efficiency.

#### Molecular genetic methods

To construct restriction-deficient mutants of *S. epidermidis* 17-20 wild type (Δ*sau3AIR*; Δ*hsdR*; Δ*sau3AIR*Δ*hsdR*), the temperature-sensitive knockout plasmid pBASE6 was used as previously described.^70^ In brief, 1-kb genomic regions directly upstream and downstream of the gene hsdR were amplified using the primers KO_hsdR_Up_fwd/KO_hsdR_Up_rev or KO_hsdR_Down_fwd/KO_hsdR_Down_rev, respectively, and digested with the restriction enzymes (Thermo Scientific) indicated in Table S4. Fragments were ligated into equally digested pBASE6 using T4-ligase (Thermo Scientific) and introduced into *E. coli* DC10B. The correct assembly of the plasmid, named pBASE6_KO_hsdR, was confirmed via PCR and sequencing. Subsequently, the plasmid was introduced into the intermediary host *S. aureus* PS187ΔΔ by standard electroporation as previously described.^41^ The resulting strain *S. aureus* PS187ΔΔ pBASE6_KO_hsdR was used generate a lysate of phage Φ187. The phage lysate of Φ187 was used for transduction of *S. epidermidis* 1457. Subsequently, lysates of ΦE72 were generated using *S. epidermidis* 1457 pBASE6_KO_hsdR. Plasmids were then introduced via heat-shock facilitated transduction into *S. epidermidis* 17-20 wild type using this phage ΦE72 lysate. The knockout procedure via homologous recombination was performed as previously described, and successful knockout was confirmed via PCR and sequencing using primers KO_hsdR_control_fwd and KO_hsdR_control_rev.^70^

For the construction of *S. epidermidis* 17-20 Δ*sau3AIR* and Δ*hsdR*Δ*sau3AIR*, the 1-kb upstream and downstream genomic regions of the sau3AIR gene, were amplified using primers KO_sau3AIR_Up_fwd/KO_sau3AIR_Up_rev or KO_sau3AIR_Down_fwd/KO_sau3AIR_Down_rev, respectively. The plasmid named pBASE6_KO_sau3AIR was constructed as described above, introduced into *S. epidermidis* 17-20 wild type as well as *S. epidermidis* 17-20 Δ*hsdR* using phage ΦE72, and the homologous recombination procedure was repeated as described above. Successful knockout was confirmed via PCR and sequencing using primers KO_sau3AIR_control_fwd and KO_sau3AIR_control_rev.

#### Heat shock transduction assay

All plasmids used for the heat shock transduction experiments with phage ΦE72 were first introduced into *S. epidermidis* 1457 as intermediary host via electroporation or standard transduction using phage Φ187.^55^ The presence of the plasmids in *S. epidermidis* 1457 was confirmed, and phage ΦE72 lysates were generated as described above. For the transduction assay, recipient strains of interest were inoculated into TSB and grown overnight. The next day, 10 mL of fresh TSB were inoculated with overnight culture to OD_600_ = 0.1. The cultures were grown shaking at 160 rpm and 37°C until OD_600_ = 0.8 was reached. Five 1.5-mL sample tubes were filled with 200 μL of bacterial culture. Cells were centrifuged for one min at 11,000 x g, the supernatant was aspirated, and the pellet was resuspended in 200 μL phage buffer (4 mM CaCl_2_, 1 mM MgSO_4_, 0.1 M NaCl, 50 mM Tris-HCl, 0.1% gelatin (w/v); adjusted to pH 7.8).

One of the aliquots was incubated each at 37°C (control), 48°C, 50°C, 52°C, or 54°C for two min in a 1.5 mL thermal block (Eppendorf) without agitation. After two min of incubation, 100 μL of plasmid-containing phage lysate was added immediately. Phage–bacteria mixtures were incubated without shaking at 37°C for 10 to 15 min to allow for adhesion and transduction to occur.^71^ Mixtures were incubated for 10 to 15 min at 30°C if a temperature-sensitive plasmid was transduced. Afterward, the mixture was plated on TSA containing an appropriate antibiotic and the plates were incubated over night at 37°C or at 30°C for temperature-sensitive plasmids. If necessary, the mixture was diluted 1:10 with phage buffer before plating to allow for colony counting.

After 24h (48 h in case of cells grown at 30°C due to carriage of temperature-sensitive plasmids), colonies were enumerated, and the transduction efficiency was calculated as ‘Colony Forming Units’/’Plaque Forming Units’ (CFU/PFU) (Equation 1).

(Equation 1) $$\frac{\text{Total}\;\text{counted}\;\text{colonies}\;\left(\text{CFU}\right)}{\text{Phage}\;\text{Titer}\;\left(\frac{\text{PFU}}{\text{mL}}\right)\;*\;0.1\;\text{mL}}=\text{CFU}/\text{PFU}$$

For heat shock transduction of the coagulase-positive species *S. pseudintermedius* ED99, the same procedure was performed. In the case of the species *Listeria grayi* and *Bacillus spizizenii*, phage Φ11 was used. For experiments with phage Φ11, plasmids were introduced into *S. aureus* RN4220 via electroporation^41^ and lysates with Φ11 generated as described above. Heat-shock transduction was performed as described above for staphylococci, with the exception that the temperature range was increased to 52°C–58°C for the transduction of *Listeria grayi*.

For heat shock transduction of *Cutibacterium acnes* KPA171202, the procedure was performed with following variation. To perform experiments, phage lysate was prepared as described above incubating PAD20 phage with *C. acnes* SLST A1 strain harboring plasmid pBR9. *C. acnes* SLST A1 was transformed with pBR9 plasmid as previously described.^47^ Recipient strain *C. acnes* KPA171202 was inoculated to starting OD_600_ of 0.1 and grown at 37°C anaerobically, 110 rpm. After 24 h 1 mL of culture was spun down 1 min, 9.500 x g and the pellet resuspended in 200 μL phage buffer (4 mM CaCl_2_, 1 mM MgSO_4_, 0.1 M NaCl, 50 mM Tris-HCl, 0.1% gelatin (w/v); adjusted to pH 7.8). Heat shock transduction was performed in triplicates at 37°C (control), 48°C, 50°C, 52°C, 54°C and 56°C for two min in a 1.5 mL thermal block (Eppendorf) without agitation. After two min of incubation, 100 μL of plasmid-containing phage lysate was added immediately. Phage–bacteria mixtures were incubated without shaking at 37°C for 24h to allow for adhesion and transduction to occur. Afterward, the mixture was plated on Brucella agar plates containing 10 μg mL^−1^ erythromycin and the plates were incubated for 7 days, anaerobically at 37°C. Colonies were enumerated, and the transduction efficiency was calculated as ‘Colony Forming Units’/’Plaque Forming Units’ (CFU/PFU) (Equation 1).

#### Cell viability assay

Competent cells of *S. aureus* RN4220 were prepared as previously described. In brief, *S. aureus* RN4220 was inoculated into 10 mL TSB and grown shaking over night at 37°C. The next day, 100 mL fresh TSB were inoculated 1:100 with the overnight culture and grown shaking at 37°C until OD_600_ = 0.5 was reached. The culture was immediately centrifuged in two aliquots of 50 mL (4,700 x g at 4°C) for 10 min to pellet the cells. Each aliquot was washed three times with ice-cold 10% glycerol (50 mL, 40 mL, 25 mL), bevor both aliquots were resuspended in a total volume of 1 mL 10% glycerol. Cells were immediately frozen at −80°C in 70 μL aliquots.

For cell viability determination aliquots were thawed on ice for 10 min 10 μL of each aliquot was used for dilution series with PBS and the dilutions plated on TSA to determine the number of viable cells without treatment. 50 μL were used to determine the effects of heat treatment, electroporation or both on the viability of the cells. For the heat shock, aliquots were incubated at 52°C for 2 min prior to dilution in PBS and plating on TSA. For electroporation, cells were transferred to 1mm electroporation cuvettes and electroporated at standard conditions (21kV ∗ cm^−1^, 100Ω, 25μFD) prior to dilution in PBS and plating on TSA. For the combined treatment, cells were first heat shocked at 52°C for two min bevor electroporation, subsequent dilution in PBS and plating on TSA. Plates were incubated over night at 37°C and the number of colonies (viable cells) determined as CFU ∗ mL^−1^ after 24 h.

#### Transformation - Transduction efficiency comparison

To prepare plasmid DNA for transformation and a lysate of phage Φ11 for transduction, *S. aureus* RN4220 carrying plasmid pRB474 was inoculated into 20 mL fresh TSB + CM10 and grown over night at 37°C. The next day, 10 mL fresh TSB + CM10 were inoculated 1:100 with the overnight culture. A lysate of *S. aureus* RN4220 with pRB474, using phage Φ11, was prepared as described above and stored at 4°C until use. The remaining overnight culture was used for plasmid preparation using the Qiagen Plasmid Midi Kit following manufacturer’s instructions with minor modifications. In brief, cells were centrifuged for 10 min at 4°C and 4,700 x g to pellet the cells. The pellet was resuspended in 4 mL _dd_H_2_0 and 50 μL lysostaphin (1mg ∗ mL^−1^; Sigma-Aldrich) were added. The resuspended cells were incubated at 37°C until the mixture appeared viscous. The remaining preparation was carried out according to the manufacturer’s protocol.

To determine transformation efficiency, 50 μL of electrocompetent *S. aureus* RN4220 WT, prepared as described above, were electroporated with 500 ng of plasmid pRB474. After electroporation, 900 μL of TSB were added to the cells and the mixture allowed to regenerate for 2 h, shaking at 37°C. After regeneration, cells were plated on TSA + CM10. After incubation at 37°C for 24 h the total number of transformants was counted. To calculate the efficiency in the form of ‘Clones per plasmid copy’, the number of plasmid DNA copies equivalent to 500 ng was calculated using the New England Biolabs (NEB) ‘dsDNA: Mass to/from Moles Calculator’, using the DNA sequence of plasmid pRB474.

Transduction of *S. aureus* RN4220 with phage Φ11, carrying plasmid pRB474, was carried out as described above without the heat shock. After transduction, the cells were plated on TSA + CM10 plates and incubated for 24 h at 37°C before the transductants were enumerated. To calculate the efficiency (‘Clones per plasmid copy’) we determined the phage titer of the lysate used for transduction as described above, using the standard plaque forming assay. It has previously been reported for phage Φ11 that about 1 in 700 phages carry mispackaged plasmid (transducing particles) instead of the phage genome.^72^^,^^73^ Using the determined phage titer, we calculated the number of transducing particles (plasmid DNA copy number) in the lysate by Equation 2.

(Equation 2) $$\frac{\text{Phage}\;\text{titre}\;\left(\frac{\text{PFU}}{\text{mL}}\right)}{700}=\frac{\text{Number}\;\text{transducing}\;\text{particles}}{\text{mL}}*0.1\;\text{mL}=\frac{\text{plasmid}\;\text{DNA}\;\text{copy}\;\text{number}}{100\;\mu L\;\text{phage}\;\text{lysate}}$$

To determine the efficiencies of both methods, the number of transformants/transductants per plasmid copy was calculated using Equation 3.

(Equation 3) $$\frac{\text{Number}\;\text{of}\;\text{transformants}\setminus \text{transductants}}{\text{Used}\;\text{plasmid}\;\text{DNA}\;\text{copy}\;\text{number}}=\text{Clones}\;\text{per}\;\text{plasmid}\;\text{copy}$$

#### Heat shock recovery assay

The assay was performed identically as the heat shock transduction assay described above; the heat shock was applied to the cells for two min as well. After incubation of the cells at 50°C for two min, 800 μL of TSB was added to the cells, and the tubes were incubated at 37°C, shaking at 160 rpm for regeneration. At t = 0, 5, 15, 30 and 60 min of incubation, individual aliquots were centrifuged one min at 11,000 x g. The supernatant was discarded, and the pellet was resuspended in 200 μL phage buffer. 100 μL of ΦE72 phage lysate containing the plasmid of interest was added, and the mixture was incubated without shaking at 37°C for 10 to 15 min, as described above. Afterward, the mixture was plated on TSA containing 10 μg/mL chloramphenicol and the plates were incubated over night at 37°C. If necessary, the mixture was diluted 1:10 with phage buffer before plating to facilitate colony counting. Transduction efficiency as CFU/PFU was calculated as above using Equation 1.

#### Bacterial genome assembly and DNA sequencing

For the assembly of bacterial genomes DNA isolation, library preparation and sequencing were performed in cooperation with the Institute for Medical Microbiology (part of the NGS Competence Center Tübingen (NCCT), Germany). Genomic DNA (gDNA) extraction was done using the Qiagen 20/G genomic tip kit, according to the manufacturer’s instructions. gDNA quantification was performed via the Qubit double-strand DNA (dsDNA) broad-range (BR) assay kit (Thermo Fisher). The preparation of the Oxford Nanopore Technologies (ONT) library was performed following the instruction manual. Native barcoding of genomic DNA (with EXP-NBD196 and SQK-LSK109; Oxford Nanopore) was performed, using 250 ng DNA as an input. Twelve microliters of template DNA were supplemented with the necessary reagents from the NEBNext Ultra II end repair/dA tailing kit (E7546S; New England Biolabs [NEB]) and the reaction was incubated at 20°C for 5 min and then at 65°C for 5 min. Subsequently, 3 mL of nuclease-free water, 0.75 mL end-prepped DNA, 1 mL native barcode (native barcoding expansion 96; EXP-NBD196), and 5 mL blunt/TA ligase master mix (M0367; NEB) were combined for barcode ligation in a new reaction vessel and incubated for 20 min at room temperature. After addition of one microliter 0.5 M EDTA, samples were pooled in a new reaction tube. Purification of the pooled samples was done using AMPure XP beads (Agencourt), everything washed twice with 70% ethanol and resuspended in nuclease-free water. For the ligation of the barcodes, 5mL adapter mix II, 10mL NEBNext quick ligation reaction buffer, and 5mL quick T4 DNA ligase were added to the pool and incubated for 10 min at room temperature. Subsequent purification was performed using AMPure XP beads, washed twice with long fragment buffer, and eluted with elution buffer. The library pool was analyzed on a MinION device (ONT) and data acquisition stopped at 39 Gb output. Base calling was performed using ONT’s Guppy base caller version 4.1.1. For Illumina short-read sequencing, libraries were prepared using the “Illumina Nextera DNA Flex library preparation kit” (Illumina) with “IDT for Illumina DNA/RNA UD indexes, Tagmentation” (Illumina) according to the manufacturer’s instructions with an input of 500 ng DNA, and 5 cycles of indexing PCR. Correct fragment length of the libraries was confirmed on an Agilent 2100 Bioanalyzer, pooled equimolarly, and quantified with a Qubit DNA high sensitivity (HS) assay kit (Thermo Fisher). The pooled libraries were sequenced on a MiSeq reagent kit v2 (300 cycles) flow cell (Illumina) with 2 x 150 bp read length. For the demultiplexing step, bcl2fastq v2.19.0.316 was used. Unicycler v0.5.0^74^ with default parameters was used for a hybrid assembly of the Oxford Nanopore and Illumina reads of the *Staphylococcus epidermidis* 17-20 (Accession number CP186575) and D2-30 genomes (Accession number CP185372). The resulting genome were annotated using prokka v1.14.6^75^ with the additional parameters to add gene features in the annotation and searching for non-coding RNAs (parameters --addgenes and --rfam). The quality of the assemblies was assessed using quast v5.3.0.^76^ Final species identification was performed via the MLST 2.0 web-application using the fasta files of the previously assembled genomes.^77^

#### DNA defense system identification

The Procaryotic Antiviral Defense LOCator (PADLOC; v2.0.0 using PADLOC-DB v2.0.0)^38^ was used to identify potential restriction systems encoded in the genomes of *S. epidermidis* 17-20, D2-30 (Accession numbers CP186575 and CP185372, respectively) and *S. pseudintermedius* ED99 (Accession number CP002478.1). Assembled genomes of *S. epidermidis* D2-30 and 17–20 were saved in fasta format. Genome sequence of *S. pseudintermedius* ED99 was downloaded from NCBI database in fasta format. Genomes were uploaded to PADLOC webserver and restriction systems evaluated on the site. All searches were performed with the ‘CRISPRDetect’ function enabled to find any potential CRISPR arrays. Confirmation of the identified RM systems was accomplished using REBASE^39^ while CRISPR-Cas systems were checked via nucleotide and protein BLAST.^78^

#### Statistical analysis

All statistical analyses were performed using GraphPad Prism version 10.0.0. Data were collected in a column table and analyzed via One-Way analysis-of-variance (ANOVA) with standard parameters. Multiple comparisons were set to the control column and corrected using Dunnett’s correction. All data were analyzed without any form of transformation and are only shown on a logarithmic scale to improve visualisation. Output style for all analyses using ANOVA were set to ‘GP’ with the following *p*-values: *p* ≥ 0.05 = ns = not significant; ∗ = *p* < 0.05; ∗∗ = *p* < 0.01; ∗∗∗ = *p* < 0.001; ∗∗∗∗ = *p* < 0.0001. For comparing the efficiencies of transformation vs. transduction, an unpaired Student’s *t* test was performed. The output style for significances was set to ‘GP’ with the following *p*-values: *p* ≥ 0.1234 = ns = not significant; ∗ = *p* < 0.0332; ∗∗ = *p* < 0.0021; ∗∗∗ = *p* < 0.0002; ∗∗∗∗ = *p* < 0.00001.

For all transduction assays with data shown as ‘transductants per PFU’, 10^−9^ was defined as limit of detection (LOD) and was defined as zero, as suggested by GraphPad.

## Supplemental information

Document S1. Figures S1–S3 and Tables S1–S4

Document S2. Article plus supplemental information
