# Supplementary material for: Genetic modification of intractable bacterial clones by heat shock-facilitated phage transduction
Source: Cell Rep Methods. 2026 Apr 20;6(5):101406. doi: 10.1016/j.crmeth.2026.101406 (PMC13198106; doi:10.1016/j.crmeth.2026.101406)
Supplement: Document S1. Figures S1–S3 and Tables S1–S4 [file mmc1.pdf]

Cell Reports Methods, Volume *6*

Supplemental information

Genetic modification of intractable bacterial clones by heat shock-facilitated phage transduction

Lukas Schulze, Jens Stahl, Nastassia J. Knödlseder, Sophia Krauss, Theresa Harbig, Kay Nieselt, Holger Brüggemann, Bernhard Krismer, and Andreas Peschel

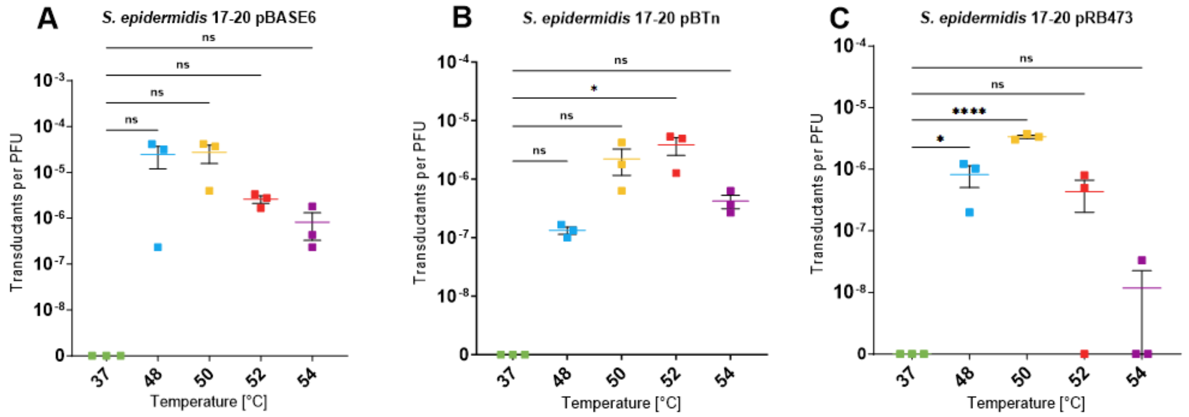

Figure S1: Heat-shock facilitated transduction of *S. epidermidis* 17-20 using different plasmids, related to Figure 2. Transductions were performed with phage ΦE72, propagated in *S. epidermidis* 1457 carrying the different plasmids. As control, cells were not heat shocked but incubated for 2 min at the regular growth temperature of 37°C. Transductants of *S. epidermidis* 17-20 per PFU shown on y-axis in logarithmic scale at the different temperatures for plasmids (**A)**, pBASE6, (**B)**, pBTn and (**C)**, pRB473. All data shown as means of three independent biological replicates (n=3) ± SEM. Statistical analysis was performed via One-Way ANOVA using Dunnett's multiple comparison test with the 37°C condition as reference. ns = not significant; *= P < 0.05; **** = P < 0.0001.

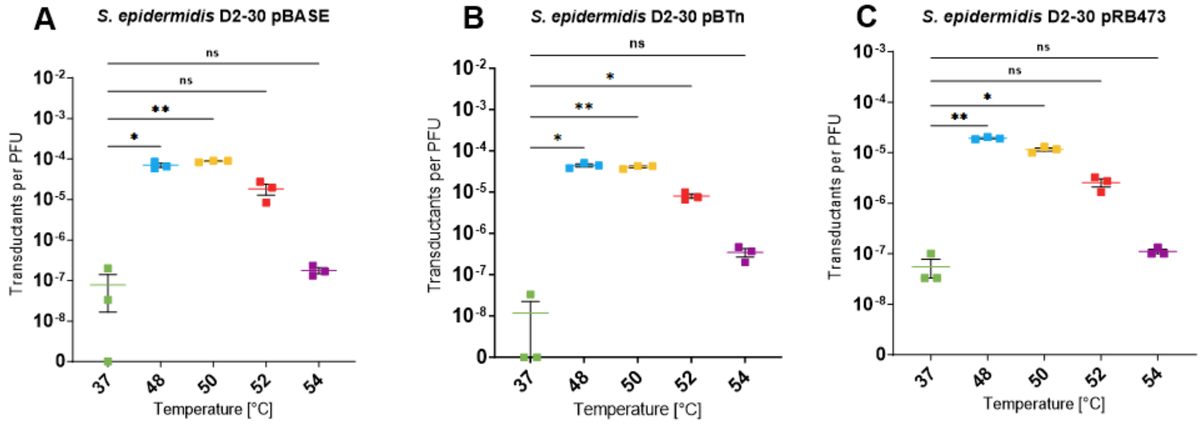

Figure S2: Heat-shock facilitated transduction of *S. epidermidis* D2-30 using different plasmids, related to Figure 2. Transductions were performed with phage ΦE72, propagated in *S. epidermidis* 1457 carrying the different plasmids. As control, cells were not heat shocked but incubated for 2 min at the regular growth temperature of 37°C. Transductants of *S. epidermidis* D2-30 per PFU shown on y-axis in logarithmic scale at the different temperatures for plasmids (**A)**, pBASE6, (**B)**, pBTn and (**C)**, pRB473. All data are shown as means of three independent biological replicates (n=3) ± SEM. Statistical analysis was performed via One-Way ANOVA using Dunnett's multiple comparison test with the 37°C condition as reference. ns = not significant; *= P < 0.05; ** = P < 0.01.

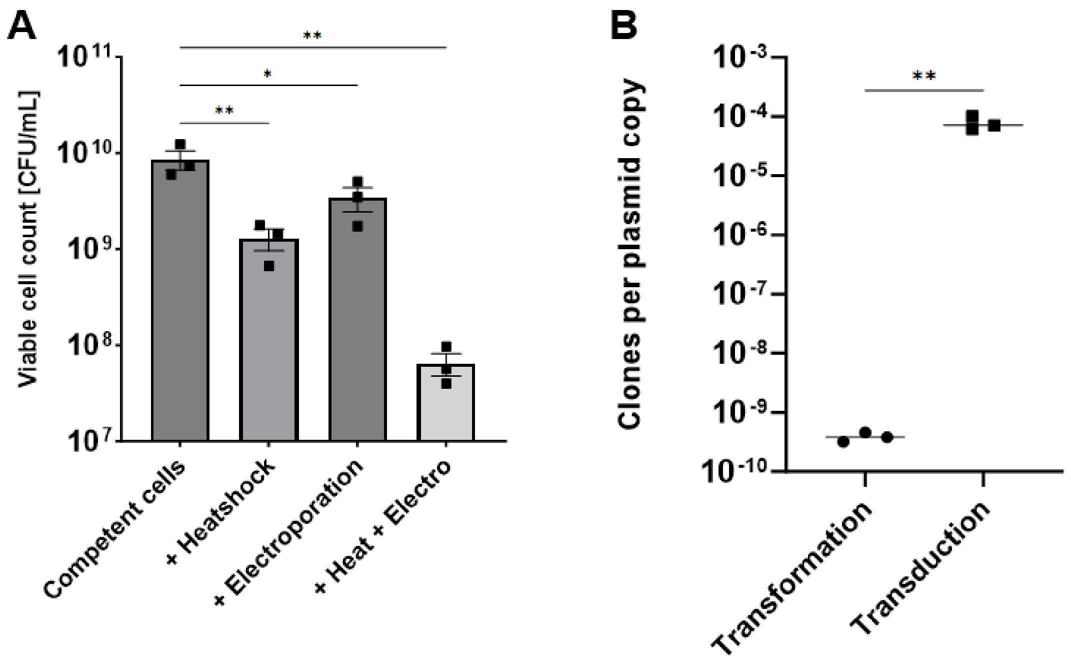

Figure S3: Viability testing and efficiency comparison of transformation vs. transduction, related to STAR Methods 'Cell viability assay' and 'Transformation-Transduction efficiency comparison' as well as results section 'Transduction shows enhanced efficiency in comparison to transformation by electroporation'. (**A**), Determination of the number of viable cells in preparations of *S. aureus* RN4220 competent cells without addition of plasmid DNA. Viability was determined in cells without any additional treatment, after heat shock for 2 min at 52°C, after electroporation (21kV * cm^{-1}, 100Ω, 25µFD) or a combined treatment with both methods. Shown are the means of three independent biological replicates (n=3) ± SEM. Statistical analysis was performed via One-Way ANOVA using Dunnett's multiple comparison test (Competent cells w/o treatment as reference). ns = not significant; *= P < 0.05; ** = P < 0.01. (**B**), Transformation and transduction efficiencies in *S. aureus* RN4220 shown as generated clones (transformants/transductants) per provided plasmid copy. Phage Φ11 was used for the transduction and was propagated on *S. aureus* RN4220 carrying plasmid pRB474. Plasmid pRB474 used for transformation was prepared from *S. aureus* RN4220. Data shown as the mean of three independent biological replicates (n=3) ± SEM. Statistical analysis was performed using unpaired student's t-test. ** = P < 0.0021.

Table S1: Bacterial species and bacteriophages used throughout this work, related to STAR Methods.

**Bacterial Species**	**Description and Source**
Bacillus spizizenii W23	DSMZ strain (DSM 8439).
Escherichia coli DC10B	Δ*dcm* mutant of cloning strain *E. coli* DH10B. [1]
Listeria grayi	Strain collection AG Peschel, patient isolate.
Staphylococcus aureus PS187ΔΔ	Restriction-deficient variant (Δ*hsdR*Δ*sauUSI*) of *S. aureus* PS187. [2]
Staphylococcus aureus RN4220	Restriction-deficient *S. aureus* strain generated by chemical and UV mutagenesis. [3]
Staphylococcus epidermidis 1457	Laboratory strain of *S. epidermidis* often used as intermediary cloning host. [4]
Staphylococcus epidermidis D2-30	Nasal isolate of *S. epidermidis*. This study.
Staphylococcus epidermidis 17-20	Nasal isolate of *S. epidermidis*. This study.
Staphylococcus epidermidis 17-20 Δ*hsdR*	Nasal isolate of *S. epidermidis,* deficient in restriction endonuclease *hsdR*. This study.
Staphylococcus epidermidis 17-20 Δ*sau3AIR*	Nasal isolate of *S. epidermidis,* deficient in restriction endonuclease *sau3AIR*. This study.

Staphylococcus epidermidis 17-20 Δ*hsdR*Δ*sau3AIR*	Nasal isolate of *S. epidermidis,* deficient in restriction endonucleases *hsdR* and *sau3AIR*. This study.
Staphylococcus pseudintermedius ED99	Clinical isolate of *S. pseudintermedius*. [5]
Cutibacterium acnes KPA171202	DSMZ strain (DSM 16379), proficient in RM system IIIB. This study.
Cutibacterium acnes A1	Skin isolate of *Cutibacterium acnes*, deficient in RM system IIIB. This study.
**Bacteriophages**	**Propagation strain and source**
Φ11	*S. aureus* RN4220. [6]
Φ187	*S. aureus* PS187ΔΔ. [7,8]
ΦE72	*S. epidermidis* 1457. [9]
PAD20 [10]	*C. acnes* skin isolate SLST A1

Table S2: Overview of RM systems identified in *S. epidermidis* D2-30, 17-20 and *S. pseudintermedius* ED99, related to Figure 1. Shown are the system type, protein name, target description, and genomic location. All information was derived from the PADLOC webserver.

***S. epidermidis* 17-20 (Accession number CP186575)**				
**System-type**	**protein.name**	**target.description**	**start**	**end**
RM_type_I	REase_I	ID=1_42;partial=00;start_type=ATG;rbs_motif=GGA/GAG/AGG;rbs_spacer=5-10bp;gc_cont=0.313	50004	52796
RM_type_I	MTase_I	ID=1_44;partial=00;start_type=ATG;rbs_motif=AGGAG;rbs_spacer=5-10bp;gc_cont=0.342	53470	55026
RM_type_I	Specificity_I	ID=1_45;partial=00;start_type=ATG;rbs_motif=AGGAG;rbs_spacer=5-10bp;gc_cont=0.267	55019	56110
RM_type_II	REase_II	ID=1_437;partial=00;start_type=ATG;rbs_motif=AGGA;rbs_spacer=5-10bp;gc_cont=0.292	504654	506120
RM_type_II	MTase_II	ID=1_438;partial=00;start_type=TTG;rbs_motif=AGGAG;rbs_spacer=5-10bp;gc_cont=0.299	506219	507457
***S. epidermidis* D2-30 (Accession number CP185372)**				
**System-type**	**protein.name**	**target.description**	**start**	**end**
DMS_other	REase_I	ID=1_30;partial=00;start_type=ATG;rbs_motif=GGAG/GAGG;rbs_spacer=5-10bp;gc_cont=0.356	40479	43598
DMS_other	Specificity_I	ID=1_31;partial=00;start_type=ATG;rbs_motif=GGAG/GAGG;rbs_spacer=5-10bp;gc_cont=0.292	43582	44838
DMS_other	MTase_II	ID=1_33;partial=00;start_type=ATG;rbs_motif=GGAG/GAGG;rbs_spacer=5-10bp;gc_cont=0.357	45400	46329
RM_type_II	REase_II	ID=1_405;partial=00;start_type=ATG;rbs_motif=AGGA;rbs_spacer=5-10bp;gc_cont=0.290	476064	477530
RM_type_II	MTase_II	ID=1_406;partial=00;start_type=TTG;rbs_motif=AGGAG;rbs_spacer=5-10bp;gc_cont=0.301	477629	478867
***S. pseudintermedius* ED99 (Accession number CP002478.1)**				
**System-type**	**protein.name**	**target.description**	**start**	**end**
RM_type_II	MTase_II	-	33256	35017
RM_type_II	REase_II	-	35075	35966
RM_type_IV	mREase_IV	-	273636	276501
cas_type_II-A	Cas9	-	677896	681901

cas_type_II-A	Cas1_II	-	681890	682766
cas_type_II-A	Cas2_II	-	682846	683140
cas_type_II-A	Csn2	-	683136	683985
CRISPR_array	CRISPR_array	CRISPR001; repeat=GTTTTAGCACTATGTTTATTTAGAAAGAGGTAAAAC;score=6.20	684166	685710

Table S3: Overview of plasmids used in this study, related to STAR Methods.

**Plasmid Size**	**Antibiotic resistance**	**Function**
pBASE6<br>6.6 kbp	Ampicillin<br>Chloramphenicol	Temperature-sensitive in staphylococci. Used for knockout via homologous recombination. [11]
pBTn<br>11.25 kbp	Chloramphenicol<br>Erythromycin	Plasmid carrying the Himar-1 transposase and a transposable erythromycin cassette for generation of transposon libraries [12]
pRB473<br>5.7 kbp	Ampicillin<br>Chloramphenicol	*E. coli* – *S. aureus* shuttle vector. [13]
pRB474<br>5.8 kbp	Ampicillin<br>Chloramphenicol	*E. coli* – *S. aureus* shuttle vector. Derivative of plasmid pRB374 that contains the *Bacillus veg* promoter for constitutive gene expression. [14]
pT183<br>4.4 kbp	Tetracyline	Derivative of plasmid pTX15 [15] that was constructed similarly as described for pC183 [16]. In brief, the xylose-inducible repressor *xylR* and the lipase gene (*geh*) were replaced by a promoter-less *gfp* and a preceding multiple cloning site using described restriction enzymes.
pBR9<br>8.5 kbp	Ampicillin<br>Chloramphenicol<br>Erythromycin	*E. coli* – *C. acnes* shuttle vector. Derivative of pBRESP36A [17] that contains the *C. acnes* p1340 promoter for constitutive expression.

Table S4: Primers used in this work, related to STAR Methods. If present, cleavage sites for restriction enzymes are written in capital letters and are highlighted in red. The corresponding enzyme indicated in 'Function and restriction site'.

**Primer Name**	**5' → 3' sequence**	**Function and restriction site**
KO_hsdR_Up_fwd	gataGGTACCgtattagctacattattaga	Amplify 1 kb upstream of *hsdR* gene. Acc65I cleavage site in UP_fwd primer.
KO_hsdR_Up_rev	gtccAAGCTTcatccattcatccacc	Amplify 1 kb upstream of *hsdR* gene. HindIII cleavage site in Up_rev primer
KO_hsdR_Down_fwd	gatcAAGCTTtaaactttataaaaatcctt	Amplify 1 kb downstream of the *hsdR*. Down_fwd primer has HindIII cleavage site.
KO_hsdR_Down_rev	gacaGTCGACcgtttgagtttcaatttt	Amplify 1 kb downstream of the *hsdR* gene. Down_fwd primer has HindIII cleavage site.
KO_hsdR_control_fwd	caggcacttacgtgtatag	Control primers to check if KO was successful via cPCR and sequencing.
KO_hsdR_control_rev	gaactccatcatacgttgat	Control primers to check if KO was successful via cPCR and sequencing.

KO_sau3AIR_Up_fwd	gataGGTACCctcttgctcacacatatg	Amplify 1 kb upstream of *sau3AIR* gene. Acc65I cleavage site in UP_fwd primer.
KO_sau3AIR_Up_rev	gtccAAGCTTcatattccatcatccttatt	Amplify 1kb upstream of *sau3AIR* gene. HindIII cleavage site in Up_rev primer
KO_sau3AIR_Down_fwd	gactAAGCTTtaggctatttatgttacaat	Amplify 1 kb downstream of the *sau3AIR* gene. Down_fwd primer has a HindIII cleavage site.
KO_sau3AIR_Down_rev	gacaGTCGACcttttaatctttttaggacc	Amplify 1 kb downstream of the sau3AIR gene. SalI restriction site was introduced in Down_rev primer.
KO_sau3AIR_control_fwd	gatattgtttgtctaataaattc	Control primers to check if KO was successful via cPCR and sequencing.
KO_sau3AIR_control_rev	ggataagtattttgaataaatc	Control primers to check if KO was successful via cPCR and sequencing.
pBR9_fwd	GTGCGAGTTCGACCTTCTGG	Control primer to confirm presence of plasmid pBR9.
pBR9_rev	TTAGTTACCCGGTTGAGCCATG	Control primer to confirm presence of plasmid pBR9.

Supplementary References

1. Monk, I.R., Shah, I.M., Xu, M., Tan, M.W., and Foster, T.J. (2012). Transforming the untransformable: application of direct transformation to manipulate genetically Staphylococcus aureus and Staphylococcus epidermidis. mBio *3*. 10.1128/mBio.00277-11.
2. Winstel, V., Liang, C., Sanchez-Carballo, P., Steglich, M., Munar, M., Bröker, B.M., Penadés, J.R., Nübel, U., Holst, O., Dandekar, T., et al. (2013). Wall teichoic acid structure governs horizontal gene transfer between major bacterial pathogens. Nature Communications *4*, 2345. 10.1038/ncomms3345.
3. Kreiswirth, B.N., Löfdahl, S., Betley, M.J., O'Reilly, M., Schlievert, P.M., Bergdoll, M.S., and Novick, R.P. (1983). The toxic shock syndrome exotoxin structural gene is not detectably transmitted by a prophage. Nature *305*, 709–712. 10.1038/305709a0.
4. Mack, D., Siemssen, N., and Laufs, R. (1992). Parallel induction by glucose of adherence and a polysaccharide antigen specific for plastic-adherent Staphylococcus epidermidis: evidence for functional relation to intercellular adhesion. Infect Immun *60*, 2048–2057. 10.1128/iai.60.5.2048-2057.1992.
5. Ben Zakour, N.L., Bannoehr, J., van den Broek, A.H., Thoday, K.L., and Fitzgerald, J.R. (2011). Complete genome sequence of the canine pathogen Staphylococcus pseudintermedius. J Bacteriol *193*, 2363–2364. 10.1128/jb.00137-11.
6. Novick, R. (1967). Properties of a cryptic high-frequency transducing phage in Staphylococcus aureus. Virology *33*, 155–166. https://doi.org/10.1016/0042-6822(67)90105-5.
7. Asheshov, E.A., and Jevons, M.P. (1963). The Effect of Heat on the Ability of a Host Strain to Support the Growth of a Staphylococcus Phage. Microbiology *31*, 97–107. https://doi.org/10.1099/00221287-31-1-97.
8. Rosenblum, E.D., and Tyrone, S. (1964). SEROLOGY, DENSITY, AND MORPHOLOGY OF STAPHYLOCOCCAL PHAGES. J Bacteriol *88*, 1737–1742. 10.1128/jb.88.6.1737-1742.1964.
9. Fišarová, L., Botka, T., Du, X., Mašlaňová, I., Bárdy, P., Pantůček, R., Benešík, M., Roudnický, P., Winstel, V., Larsen, J., et al. (2021). Staphylococcus epidermidis Phages Transduce Antimicrobial Resistance Plasmids and Mobilize Chromosomal Islands. mSphere *6*, 10.1128/msphere.00223–00221. 10.1128/msphere.00223-21.
10. Lood, R., and Collin, M. (2011). Characterization and genome sequencing of two Propionibacterium acnes phages displaying pseudolysogeny. BMC Genomics *12*, 198. 10.1186/1471-2164-12-198.
11. Geiger, T., Francois, P., Liebeke, M., Fraunholz, M., Goerke, C., Krismer, B., Schrenzel, J., Lalk, M., and Wolz, C. (2012). The Stringent Response of Staphylococcus aureus and Its Impact on Survival after Phagocytosis through the Induction of Intracellular PSMs Expression. PLOS Pathogens *8*, e1003016. 10.1371/journal.ppat.1003016.
12. Li, M., Rigby, K., Lai, Y., Nair, V., Peschel, A., Schittek, B., and Otto, M. (2009). Staphylococcus aureus Mutant Screen Reveals Interaction of the Human Antimicrobial Peptide Dermcidin with Membrane Phospholipids. Antimicrobial Agents and Chemotherapy *53*, 4200–4210. 10.1128/aac.00428-09.
13. Brückner, R., Wagner, E., and Götz, F. (1993). Characterization of a sucrase gene from Staphylococcus xylosus. J Bacteriol *175*, 851–857. 10.1128/jb.175.3.851-857.1993.
14. Brückner, R. (1992). A series of shuttle vectors for Bacillus subtilis and Escherichia coli. Gene *122*, 187–192. https://doi.org/10.1016/0378-1119(92)90048-T.
15. Peschel, A., Ottenwälder, B., and Götz, F. (1996). Inducible production and cellular location of the epidermin biosynthetic enzyme EpiB using an improved staphylococcal

expression system. FEMS Microbiology Letters *137*, 279–284. 10.1111/j.1574-6968.1996.tb08119.x.

16. Burian, M., Rautenberg, M., Kohler, T., Fritz, M., Krismer, B., Unger, C., Hoffman, W.H., Peschel, A., Wolz, C., and Goerke, C. (2010). Temporal Expression of Adhesion Factors and Activity of Global Regulators during Establishment of Staphylococcus aureus Nasal Colonization. The Journal of Infectious Diseases *201*, 1414–1421. 10.1086/651619.

17. Jore, J.P.M., Luijk, N.v., Luiten, R.G.M., Werf, M.J.v.d., and Pouwels, P.H. (2001). Efficient Transformation System for<i>Propionibacterium freudenreichii</i> Based on a Novel Vector. Applied and Environmental Microbiology *67*, 499–503. doi:10.1128/AEM.67.2.499-503.2001.
